# Pulmonary Tuberculosis Masking Lung Adenocarcinoma: A Diagnostic Pitfall Following Apparent Treatment Response

**DOI:** 10.7759/cureus.108407

**Published:** 2026-05-07

**Authors:** Raihana Laamim, Adil Zegmout, Hicham Souhi, Hanane Elouazzani, Ismail Rhorfi

**Affiliations:** 1 Pulmonology Department, Mohammed V Military Training Hospital, Rabat, MAR

**Keywords:** atypical evolution, coexistence, lung adenocarcinoma, pulmonary tuberculosis, tuberculosis masking malignancy

## Abstract

Pulmonary tuberculosis and lung cancer may present with overlapping clinical and radiological features, making diagnosis challenging. We report the case of a 42-year-old chronic smoker with bacteriologically confirmed pulmonary tuberculosis who showed marked clinical and radiological improvement after anti-tuberculosis therapy. Within 20 days of this apparent favorable evolution, the patient developed hemoptysis associated with rapid clinical deterioration. Imaging revealed a right hilar mass with metastatic disease. Bronchoscopic findings and histopathological examination confirmed lung adenocarcinoma (TTF-1 positive). This case highlights how apparent treatment response can be misleading and may delay the recognition of an underlying malignancy. Reassessment should be considered when the clinical course becomes atypical or rapidly progressive, particularly in high-risk patients.

## Introduction

Pulmonary tuberculosis remains a common condition in countries with a high endemic burden, where it represents a major cause of prolonged respiratory symptoms. In parallel, lung cancer is the leading cause of cancer-related mortality worldwide and primarily affects smokers [1,2].

In such patients, who may present with nonspecific symptoms and overlapping radiological findings, the persistence or emergence of pulmonary abnormalities during follow-up may raise diagnostic uncertainty. This issue becomes particularly relevant when new findings occur after an initial clinical and radiological improvement, as early treatment response may support a single diagnosis without definitively excluding an underlying condition. We report a case of lung adenocarcinoma diagnosed shortly after apparent improvement of bacteriologically confirmed pulmonary tuberculosis.

## Case presentation

We report the case of a 42-year-old chronic smoker (25 pack-years) with no prior history of tuberculosis. He presented with a two-month history of right-sided pleuritic chest pain, resistant to analgesics, associated with night sweats, anorexia, and unquantified weight loss, without hemoptysis at baseline.

**Figure 1:**
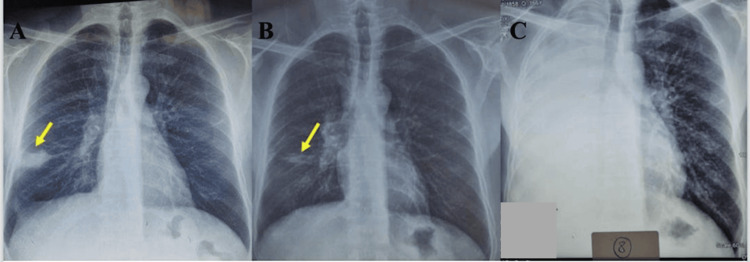
Serial chest radiographs illustrating disease progression (A) Initial radiograph showing a right mediobasal opacity (arrow). (B) Follow-up radiograph demonstrating significant regression of the lesion after anti-tuberculosis therapy (arrow). (C) Radiograph at clinical deterioration showing extensive right-sided opacification.

On admission, vital signs were within normal limits, with no fever and preserved oxygen saturation on room air. The patient was in good general condition with mild limitation due to pain. Respiratory examination showed crackles and decreased breath sounds over the right basal peripheral lung field. Chest radiography showed a well-defined homogeneous opacity in the right mediobasal region (Figure 1A).

Laboratory tests revealed elevated inflammatory markers, with leukocytosis (white blood cell count: 10,600/mm³), neutrophilia (8,000/mm³), and elevated C-reactive protein (95 mg/L). Initial sputum GeneXpert and acid-fast bacilli smears were negative. HIV serology was negative.

**Figure 2:**
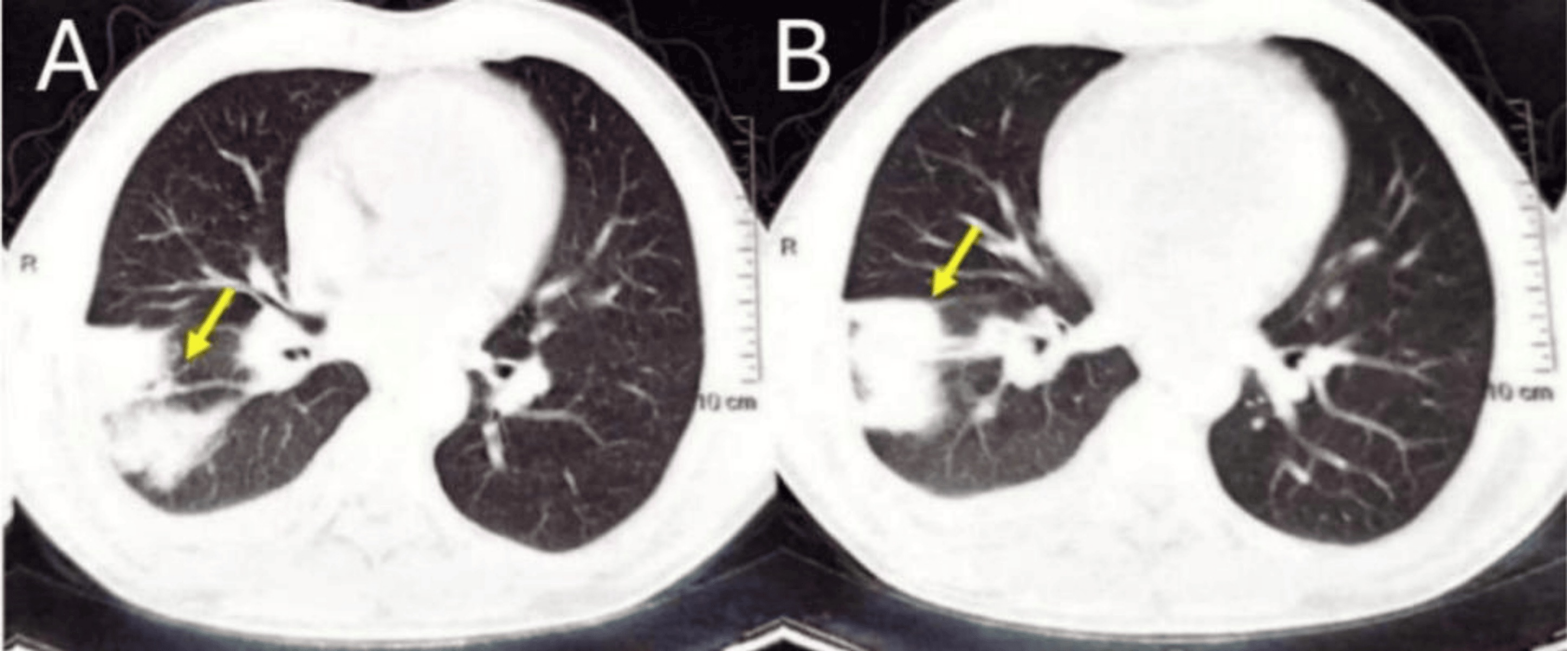
Axial chest CT images showing (A, B) right lower lobe consolidation with air bronchogram (arrows)

Chest computed tomography showed a peripheral right lower lobe consolidation in contact with the pleura, associated with centrilobular micronodules in a tree-in-bud pattern, suggestive of an infectious bronchiolar process. No definite features suggestive of malignancy were identified at this stage (Figure 2).

Flexible bronchoscopy showed no visible endobronchial lesion. GeneXpert performed on the bronchial aspirate was positive for *Mycobacterium tuberculosis*, with no rifampicin resistance. In the context of compatible clinical, radiological, and microbiological findings, pulmonary tuberculosis was considered, and standard first-line anti-tuberculosis therapy (2RHZE/4RH) was initiated.

At five months of follow-up, the patient showed clinical improvement, with a 5-kg weight gain, and marked radiological improvement, with near-complete resolution on chest radiography (Figure 1B). Twenty days later, while still on treatment, he was readmitted with mild hemoptysis and rapid clinical deterioration, with an 11-kg weight loss over a short period, while imaging showed significant disease progression (Figure 1C).

**Figure 3:**
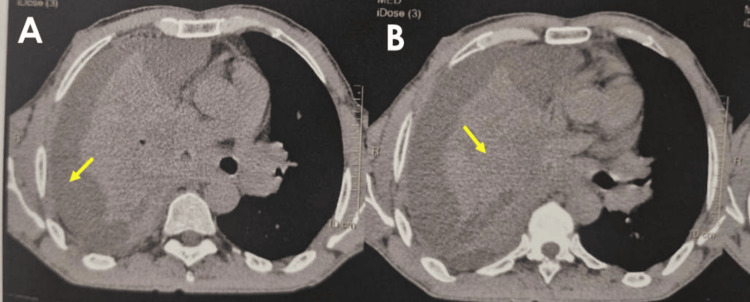
Axial chest CT images demonstrating right pleural effusion with associated atelectasis (A) Large right pleural effusion (arrow). (B) Associated atelectasis of the right lung (arrow), likely compressive in the context of bronchial obstruction.

In view of this atypical and rapidly progressive course despite appropriate therapy, further diagnostic reassessment was undertaken. Repeat chest computed tomography revealed a right hilar mass (47 × 25 mm) causing obstruction of the right main bronchus with complete lung collapse, associated with a large right pleural effusion (Figure 3), multiple bilateral heterogeneous pulmonary lesions (the largest measuring 38 × 34 mm), contralateral pulmonary nodules, mediastinal lymphadenopathy, and hepatic and adrenal lesions, suggestive of metastatic disease.

**Figure 4:**
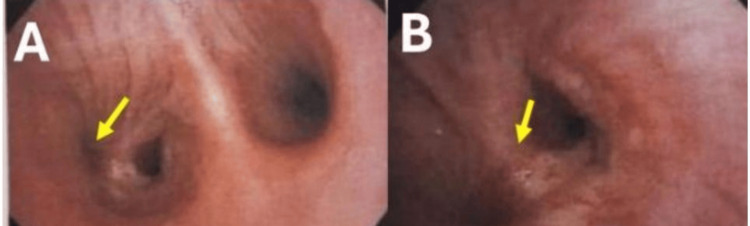
Bronchoscopic visualization of diffuse infiltrative stenosis involving the right bronchial tree (A) Severe narrowing of the right upper lobe bronchial orifice, which was non-catheterizable (arrow). (B) Diffuse infiltrative endobronchial lesion with irregular mucosa resulting in stenosis of the right bronchial tree (arrow).

Bronchoscopy revealed infiltrative stenosis of the right main bronchus, with friable mucosa and complete obstruction of the right upper lobe bronchus (Figure 4).

Histopathological examination of bronchial biopsy specimens revealed a malignant epithelial proliferation with moderate to marked cytonuclear atypia. Immunohistochemistry showed diffuse positivity for TTF-1, while P63, chromogranin, and synaptophysin were negative, supporting the diagnosis of primary pulmonary adenocarcinoma. Molecular testing for *EGFR*, *ALK*, and *ROS1* alterations was performed and showed no detectable mutations.

Overall, the findings were consistent with advanced-stage disease (T4N3M1c). The case was discussed in a multidisciplinary tumor board meeting, and platinum-based systemic chemotherapy was initiated.

## Discussion

Beyond their occasional coexistence, reported in approximately 1-2% of cases, pulmonary tuberculosis has been consistently associated with a modest increase in long-term lung cancer risk (relative risk 1.5-2), independently of smoking [3-5].

Several studies have described a complex and bidirectional relationship between pulmonary tuberculosis and lung cancer. Chronic inflammation, immune dysregulation, epithelial injury, and fibrotic remodeling induced by *Mycobacterium tuberculosis* infection have been proposed as potential mechanisms involved in bronchial carcinogenesis, particularly in smokers [5,6]. However, despite these plausible biological mechanisms, this relationship remains largely associative, and tuberculosis may coexist with or reveal a pre-existing malignancy rather than directly inducing carcinogenesis.

In most cases, the interval between tuberculosis and lung cancer diagnosis spans several months to years. The risk is markedly increased within the first two years following tuberculosis diagnosis (HR ≈ 5), then gradually declines and may disappear after four years [7]. The unusually short interval observed in our patient raises the possibility that the malignancy was already present but initially masked by inflammatory changes.

The diagnosis of lung cancer in this setting is particularly challenging due to overlapping clinical and radiological features. Although malignancy was initially considered, apparent improvement under anti-tuberculosis therapy may delay further investigations, especially in high-risk patients, as initial microbiological confirmation and favorable evolution can reinforce diagnostic anchoring.

The concomitant management of pulmonary tuberculosis and lung cancer may pose therapeutic challenges due to drug-drug interactions and cumulative toxicities. Rifampicin, a potent cytochrome P450 inducer, may reduce the efficacy of several anticancer agents, while chemotherapy can exacerbate hepatic and hematological toxicities associated with anti-tuberculosis drugs [8]. In our case, chemotherapy was initiated after completion of anti-tuberculosis treatment, which had already been completed at diagnosis, without significant adverse events.

This case has some limitations, including the absence of mycobacterial culture and histological confirmation of tuberculosis, which precludes absolute diagnostic certainty. The possibility of contamination during bronchoscopy should also be considered, although it appears unlikely given the overall clinical course.

Despite these limitations, the marked clinical and radiological response to anti-tuberculosis therapy supports the initial diagnosis, while subsequent rapid deterioration highlights the possibility of an underlying or concomitant malignancy.

This case illustrates that apparent cure of pulmonary tuberculosis may conceal an underlying lung cancer. It suggests that clinical improvement alone should not preclude early and thorough reassessment in cases of atypical or rapidly evolving disease, particularly in high-risk patients such as smokers.

## Conclusions

This case challenges the conventional perception of a temporal sequence between pulmonary tuberculosis and lung cancer, as the extremely short interval observed raises the possibility of a pre-existing malignancy. The apparent initial improvement under anti-tuberculosis therapy may have delayed further investigations, leading to the diagnosis of lung adenocarcinoma at a metastatic stage, for which systemic chemotherapy was initiated. These findings highlight the importance of maintaining clinical vigilance regarding this diagnostic overlap, particularly in tuberculosis-endemic settings, and support early reassessment in atypical cases.

## References

[1] World Health Organization (2023). Global Tuberculosis Report 2023. https://www.who.int/teams/global-programme-on-tuberculosis-and-lung-health/tb-reports/global-tuberculosis-report-2023.

[2] Sung H, Ferlay J, Siegel RL (2021). Global cancer statistics 2020: GLOBOCAN estimates of incidence and mortality worldwide for 36 cancers in 185 countries. CA Cancer J Clin.

[3] Rihawi A, Huang G, Al-Hajj A (2016). A case of tuberculosis and adenocarcinoma coexisting in the same lung lobe. Int J Mycobacteriol.

[4] Ounteini AM, Didier J, Rabiou S (2020). Second primary bronchial cancer and concomitant active pulmonary tuberculosis: coexistence of two frequent and serious respiratory diseases. J Func Vent Pulm.

[5] Brenner DR, McLaughlin JR, Hung RJ (2011). Previous lung diseases and lung cancer risk: a systematic review and meta-analysis. PLoS One.

[6] Byun HG, Yoo JY, Kim SJ, Lee OJ, Yoo MY (2019). Coexistence of lung adenocarcinoma and pulmonary tuberculosis within a single lesion: a rare case report. Medicine (Baltimore).

[7] Cabrera-Sanchez J, Cuba V, Vega V, Van der Stuyft P, Otero L (2022). Lung cancer occurrence after an episode of tuberculosis: a systematic review and meta-analysis. Eur Respir Rev.

[8] Sun M, Ji H, Liu A, Xu N (2025). Concurrent active pulmonary tuberculosis and small cell lung cancer: diagnostic challenges and therapeutic insights from a case report. Front Oncol.

